# Support in the Shadows: Findings From a Qualitative Study Exploring Fathers’ and Non‐Birthing Partners’ Perceptions and Experiences of Support During a Termination for Medical Reasons

**DOI:** 10.1111/hex.70799

**Published:** 2026-08-02

**Authors:** Sarah Fogarty, Kirstin Tindal, Alexander E. P. Heazell, Niki Munk, Frances M. Boyle, Phillipa Hay

**Affiliations:** ^1^ School of Medicine Western Sydney University Campbelltown Australia; ^2^ Centre of Research Excellence in Stillbirth, Mater Research The University of Queensland Brisbane Australia; ^3^ The Ritchie Centre Hudson Institute of Medical Research Melbourne Australia; ^4^ Department of Obstetrics and Gynaecology Monash University Melbourne Australia; ^5^ Maternal and Fetal Health Research Centre, School of Medical Sciences University of Manchester Manchester UK; ^6^ Department of Obstetrics, Saint Mary's Hospital Manchester University NHS Foundation Trust Manchester UK; ^7^ School of Health & Human Sciences Indiana University Indianapolis Indiana USA; ^8^ Massage Therapy Foundation Evanston Illinois USA; ^9^ Institute for Social Science Research The University of Queensland Brisbane Australia; ^10^ Translational Health Research Institute Western Sydney University Penrith Australia; ^11^ Mental Health Services, SWSLHD Campbelltown Hospital Campbelltown Australia

**Keywords:** fathers, non‐birthing partners, partners, termination for foetal abnormalities, termination for medical reasons

## Abstract

**Background:**

Termination for medical reasons (TFMR) is an essential aspect of reproductive healthcare. While physical and emotional care of the birthing parent is central within healthcare systems, less is known about the experiences and support needs of non‐birthing partners during this process.

**Objective:**

To explore fathers’ and non‐birthing partners’ perceptions and experiences of support (covering clinical, psychological, and social support) during a TFMR.

**Design:**

A qualitative study using semi‐structured interviews.

**Setting and Participants:**

Ten fathers/non‐birthing partners who had experienced TFMR in the last 7 years, identified via social media or snowball sampling, were interviewed.

**Analysis:**

Data were analysed using inductive thematic analysis and a mixture of semantic and latent coding of the data.

**Results:**

An overarching theme of *Support in the shadows* was identified, capturing how non‐birthing partners were consistently present but rarely the focus of explicit or sustained care or support. Three themes were developed under the main theme of *support in the shadows:* (a) Feeling invisible, (b) seeking whole‐person support and (c) partners as a support rather than being supported.

**Discussion:**

In common with other forms of pregnancy loss, fathers/non‐birthing partners described a pattern of silent responsibility, emotional invisibility and a lack of meaningful recognition of their needs. When care was individualised and specific to non‐birthing partners, it was experienced as supportive.

**Conclusions:**

The support that fathers/non‐birthing partners experienced during TFMR was largely incidental rather than embedded. Partners desired care and support that was deliberate, inclusive and individualised and covered clinical, psychological and social aspects.

**Patient or Public Contribution:**

A parent with lived experience of TFMR was involved in the study conception and methodology. The lived experience parent advised on study design and the presentation of participant information. Parents who had lived experience of TFMR in the last 7 years were interviewed for the study as participants.

## 1 Introduction

Termination for medical reasons (TFMR) is ‘when a pregnancy is terminated due to a chromosomal, genetic or structural foetal abnormality, or where continuing the pregnancy would risk the health or life of the mother’ [1]. TFMR is an essential aspect of reproductive healthcare. Like other forms of pregnancy loss, experiences are shaped not only by complex medical decision‐making but also by the personal and family circumstances in which that care is accessed [2]. Parents may endure a prolonged and distressing trajectory from the initial indication that something may be wrong, through additional testing, specialist consultations, and the termination process itself [2]. Parents who experience TFMR frequently report self‐blame and guilt associated with the termination decision [3]. This guilt and shame can increase social isolation as parents may choose not to disclose the circumstances of the loss to their social network due to the stigma surrounding TFMR [4]. In Australia, TFMR has become increasingly visible within public and political discourse, with legislative reforms shifting termination of pregnancy from a criminal to a health framework, recognising reproductive healthcare as an essential aspect of healthcare [5]. These reforms mean that termination is legal in all states and territories in Australia and is regulated as a component of healthcare [6]. While greater awareness has the potential to improve understanding, it has also been accompanied by misinformation, polarised debate and limited public appreciation of the complex medical and psychosocial circumstances in which TFMR occurs [1, 7, 8]. While policy and research have largely focused on the pregnant person's healthcare needs and access to termination services, considerably less attention has been paid to the needs of fathers and non‐birthing partners, despite their important role throughout the TFMR journey and the profound impact of pregnancy loss on the family as a whole.

Research across the broader spectrum of pregnancy loss and neonatal death demonstrates that partners often adopt the role of primary supporter and protector while navigating and managing their own grief [9, 10]. Partners report lacking resources, guidance and support needed to carry this responsibility [9] and often describe feeling neglected by healthcare professionals [10]. A recent meta‐synthesis of 18 studies involving over 300 fathers who experienced miscarriage, stillbirth and neonatal death highlighted that partners commonly experience limited support from family, friends and services, contributing to deepened isolation and reliance on personal coping strategies [11]. Men/fathers who experience TFMR report intense emotional strain including fear, anger, loneliness and a sense of exclusion [12]. Although research on men's or fathers’ grief and experience of pregnancy loss has increased [2, 12, 13, 14, 15, 16, 17, 18, 19], little research has examined their specific needs and support experiences in the context of TFMR; there are very few studies of non‐birthing partners in same‐sex or gender‐diverse relationships. One qualitative study found the decision to terminate was profoundly difficult for men as they often feel overlooked by healthcare and bereavement services, and that men valued tailored, partner‐specific support [14]. Thus, this study aimed to explore fathers’ and non‐birthing partners’ perceptions and experiences of support (covering clinical, psychological and social support) during a TFMR.

## 2 Methods

The study used qualitative in‐depth interview methods. We adopted a broad conceptualisation of support, encompassing clinical care as well as the psychological, emotional, practical, social and relational dimensions of fathers’ and non‐birthing partners’ experiences throughout the TFMR journey. The study was approved by Western Sydney University Human Ethics Committee (approval number H15715) on 8 December 2023. As part of the ethics approval, a participant distress protocol was developed to guide researchers in responding to emotional distress, including offering breaks, discontinuing the interview if requested and facilitating referral to appropriate support services where required.

### 2.1 Participants

Participants were fathers/non‐birthing partners who had experienced a TFMR in the last 7 years. A 7‐year cut‐off recognised that the healthcare landscape has changed over time, particularly with the growing attention to TFMR in Australia, meaning experiences of support from earlier periods may have been less representative of current practice. The aim was to recruit 50% of the sample who had experienced a TFMR in the last 24 months to reduce potential recall bias and maximise the relevance of participants’ experiences to reflect contemporary practice. Inclusion criteria were (a) residing in Australia, (b) being 18 years of age or greater, (c) experiencing a TFMR in the last 7 years as a non‐birthing partner and (d) being able to speak and understand English.

### 2.2 Procedure

Potential participants were approached in two ways: (1) via social media (through the first author's professional business/research Instagram and Facebook accounts) or (2) via snowball sampling through mothers who participated in another study. Potential participants completed an online expression of interest form which included the screening question ‘*have you previously experienced a termination for medical reasons in the last 7 years?* and those that met the criteria were provided with a copy of the patient information form and had the opportunity to ask questions. Data were not retained from those that answered no to the screening question. Informed consent was provided via a written, signed and dated informed consent form. At the time of the interview, consent was reconfirmed verbally for the interview to be recorded.

Demographic information was collected once participants had consented to be part of the study via an email link to complete an online questionnaire (Qualtrics). Demographic information collected included age, sex assigned at birth, country of birth, Indigenous status, geographical classification, highest level of education, marital and job status, as well as medical information about the pregnancy and TFMR.

Author S.F. conducted the qualitative online in‐depth interviews. The interview guide was adapted from the guide used in the study of mothers’ experiences of support in their TFMR journey (see Supporting Information) with only minor changes in terminology to ensure appropriateness for fathers/non‐birthing partners. The mothers’ interview guide was developed in collaboration with a parent with lived experience of TFMR. The original interview guide was not piloted, but by the time it was used for this study, 23 interviews using this guide had been completed. The interviews took place via the Zoom online platform at a time of the participants' choosing. Zoom meetings were password‐protected, enabled authenticated log‐ins (signed into a Zoom account) and not on the public setting. The link for the meeting was only sent to the person being interviewed. Interviews were recorded and transcribed. Interviews took between 90 and 120 min to complete.

### 2.3 Reflexivity Statement

All authors are researchers and parents. Five authors have personal experience of pregnancy loss (S.F./A.H./P.H./K.T./F.B.). S.F. is a massage therapist with experience in supporting parents who have experienced perinatal loss. A.H. serves as the clinical lead for a specialist pregnancy‐after‐loss clinic in the UK. P.H. is a psychiatrist with expertise counselling individuals and families after stillbirth, with a particular interest in psychotherapeutic approaches that may be more acceptable after pregnancy loss, such as alternatives to medication. N.M. is an experienced massage therapist and researcher with a focus on working with vulnerable populations. K.T. is a researcher with experience in best‐practice co‐design in pregnancy loss research and has co‐developed frameworks and resources to support bereaved parents’ involvement in pregnancy loss research. F.B. is a social scientist with a primary research focus on perinatal loss support.

### 2.4 Member Checking

Member checking [20] was provided as an option for study participants to provide feedback on the study findings. The primary aim for member checking was to invite feedback, given the narrative presentation of the findings, to help avoid errors or misinterpretations of the represented participants’ perspectives. All participants were invited via email to review and provide feedback on whether the findings and selected quotes accurately reflected their experiences.

### 2.5 Data Analysis

Demographic data were summarised using summary statistics for continuous variables (e.g., mean ± SD) and categorical variables (e.g., counts).

Interview data were analysed using reflexive thematic analysis applying a constructivist epistemology (the belief that humans create their own understanding and knowledge of the world through experiencing things and reflecting on those experiences [21]), and an inductive analysis approach and a mixture of semantic and latent coding of the data [22, 23, 24]. Authors S.F. and K.T. immersed and familiarised themselves with the data to ascertain and identify the key concepts. Consistent with reflexive thematic analysis, coding was conducted recursively rather than linearly. S.F. and K.T. moved repeatedly between the data and developing codes, reflecting on patterns of meaning and refining codes as their interpretations evolved. Following refinement of the initial codes, S.F. and K.T. examined relationships between codes, considering how individual codes connected and contributed to broader patterns of meaning across the dataset. Candidate themes were then developed by grouping related codes and organising them around central concepts that captured important aspects of the research question. These preliminary themes were reviewed, refined and revised through further engagement with the dataset to ensure they represented the complexity and diversity of participants’ experiences. Themes were subsequently defined and named to capture their central organising concepts and the underlying meanings they represented. The identified themes were discussed with members of the research team to ‘sense‐check ideas, or to explore multiple assumptions or interpretations of the data,…aiming to achieve rich interpretations of meaning’ [22, 23, 24].

### 2.6 Findings

Thirteen fathers/non‐birthing partners enquired about the study. Twelve consented to be part of the study, but two withdrew, unable to attend the interview. Ten fathers/non‐birthing partners completed the interviews. Interviews were conducted online between February and June 2024. Data collection was concluded after the 10th interview. This stopping point was determined by pragmatic considerations regarding participant recruitment and project timelines, rather than an indication of a theoretical theory as qualitative analysis only occurred after data collection was concluded. The initial findings were sent to all participants, and no participants provided feedback.

Participants had a mean age of 36.2 years, and most were male, born in Australia, lived in metropolitan areas/major cities, were married or partnered, and were engaged in full‐time employment (Table 1). Seven participants resided in Australia's two most populous states (New South Wales and Victoria). Four fathers/non‐birthing participants had partners who participated in the mothers/birthing partners arm of the study (unpublished).

**Table 1:** Participant socio‐demographic features.

Participants (*n* = 10)	Distribution
*Age*	
(Mean, SD)	36.2 years (+/−5.7)
	*n*
*Sex assigned at birth*	
Male	8
Female/prefer not to say	2
*Country of birth*	
Australia	8
Other	2
*Aboriginal or Torres Strait Islander origin*	
No	10
*Modified Monash Model geographical classification system* [25] *of primary place of work*	
MMI (Metropolitan area/Major cities)	10
*Highest level of education*	
Non‐tertiary education, e.g., Year 12 only, Certificate III/IV, Advanced Diploma/Diploma,	6
Tertiary education, e.g., Bachelor's degree, Graduate Diploma/Graduate Certificate, Postgraduate Degree	4
*Marital status*	
Married/partnered	10
*Job status*	
Employed full time or part time	6
Self‐employed full time	4

Most participants (*n* = 7) had one or more living children at the time of the interview, and most had not experienced any other pregnancy loss (*n* = 7). At the time of completing the demographic data, the participants’ TFMR was on average, 5 years and 1 month ago (+/−4 years and 2 and a half months). The average gestation at the time of the TFMR was 22 weeks and 6 days (+/−4 weeks and 1 day). All participants had been expecting a singleton, and most babies were delivered vaginally (*n* = 9). Half of the participant's birthing partners had an injection to stop the baby's heartbeat, followed by medical induction of labour. For the remaining participants partners, their procedures included medical induction of labour only, having medication to end the pregnancy then medical induction of labour, or taking medicine to end the pregnancy. In three cases, the baby was born alive.

### 2.7 Qualitative Findings

#### 2.7.1 Overall Theme: Support in the Shadows

The overall theme was *support in the shadows*. This theme captures fathers’/non‐birthing partners’ experiences of support during TFMR. While fathers/non‐birthing partners were present throughout each stage of the process—from diagnosis, waiting, decision‐making, the procedure, birth and post‐birth, the support directed towards them was rarely explicit or sustained. Medical information and care were typically directed towards the birthing partner, leaving the fathers/non‐birthing partner feeling invisible and as if their needs were peripheral to those of their partner.

Beyond the healthcare setting, fathers/non‐birthing partners found limited avenues for support from community, family, friends or peers. Fathers/non‐birthing parents, however, found moments of being out of the shadows where they felt visible and validated. This occurred when clinicians or peers acknowledged them directly, when rituals included them, or when peers or healthcare workers created safe spaces for their grief. These moments reinforced their role as parents and partners, highlighting the importance of being recognised as part of the parenting dyad.

**Figure 1:**
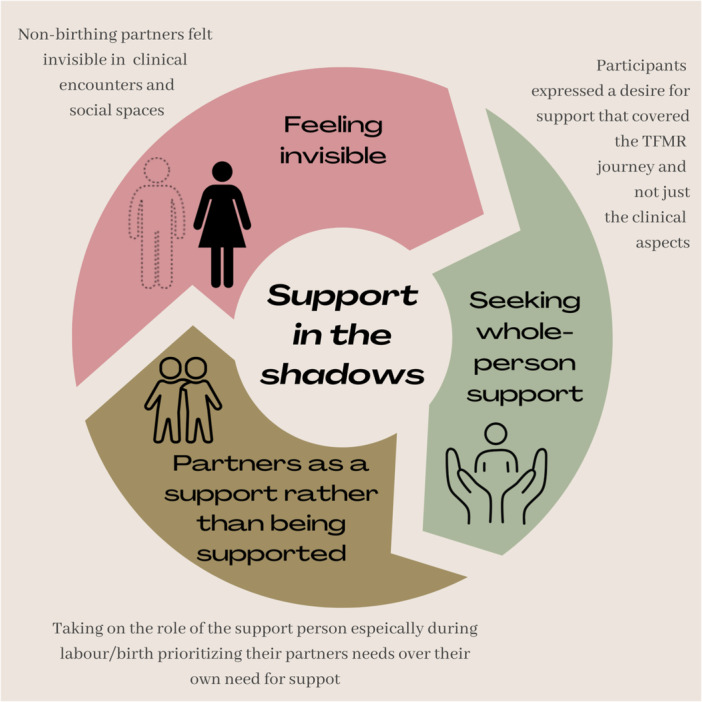
Visual representation of the overall theme and sub‐themes.

There are three themes under the main theme of *support in the shadows:* (a) Feeling invisible, (b) seeking whole‐person support and (c) partners as a support rather than being supported. See Figure 1.

a. **Feeling invisible**
  Feeling invisible encompasses the aspects of invisibility that fathers/non‐birthing parents experienced in clinical encounters and social spaces. This included instances where their needs for information, recognition and support went unacknowledged, leaving them invisible to others prioritised by the system. While there were moments where non‐birthing parents felt seen, heard and accommodated, these were infrequent.
  Participants recounted circumstances in which their presence and role as a parent were not acknowledged or seen. This could be as broad as ‘*nobody ever asked me how I feel or anything… I never felt supported’* [participant 11] or ‘*in so many instances you are not seen and not acknowledged*’ [Participant 06] to more specific circumstances in which the fathers/non‐birthing parents felt invisible to the healthcare providers and adjunct services related to pregnancy loss. This is reiterated by participant 4 who ‘*never had any direct contact from any of the medical people. It's all through my wife’*. The lack of visibility was noticeable in the community for participant 5, who experienced that ‘*any sort of conversation that sort of lead to it* [TFMR*]…it always somehow comes back around to our journey, not my journey*’.
  For some fathers/non‐birthing partners, the invisibility and lack of acknowledgement of being involved was printed on paperwork where their child was referred to as ‘baby of the birthing partner’. For one non‐birthing parent, this was etched permanently ‘*on our plaque on the urn, instead of it being* [child's first, middle and last name], *it was Baby of* [birthing partner's first and last name]*. And that was really…like, a), not even using his name, which was pretty full on, but b), like even if it's son of, it should be son of… XXX and YYY [first name of both parents]*’ (Participant 2).
  Fathers/non‐birthing parents were often not offered the same auxiliary non‐medical aspects of care as their birthing partner when they were in hospital giving birth to their child. The non‐provision of companion meals, for example, contributed to fathers’ feelings of exclusion/invisibility: ‘*I remember her* [nurse] *looking at me and going like, no, you're not the patient… I just remember thinking that moment like, whoa, like this doesn't cost anything for you to do. I'm holding my dead child in my arms. It's been the most traumatic event in my life, and you don't get fed’*. One father/non‐birthing parent was invoiced by the hospital to ‘*unfold the couch and make up a bed and for a meal that I never got*’ [Participant 12].
  Some fathers/non‐birthing partners described feeling invisible in their interactions with funeral homes: ‘*we were sitting at a round table. I sat next to* [partner]*. The funeral lady comes in and she said, honestly, if you don't want to be here, you don't have to because everything's directed at the mum anyway’* [Participant 5].
  Support needs varied among fathers/non‐birthing partners, and what was helpful for one person was not helpful for another. A common theme, however, was the lack of individualised support, specifically tailored to fathers/non‐birthing partners. Bereavement resources were perceived as largely designed for and oriented to the birthing partner, leaving fathers/non‐birthing partners feeling peripheral or not welcome users of these services. As participant 2 explained: ‘*I don't even think any of those resources were even offered to me… In my mind, they're just for the mum, or maybe a couple*’. In response, participants described seeking out their own supports, including acupuncture, hiking/running and fundraising, connecting with other fathers/non‐birthing partners, doulas and receiving ongoing follow‐up care. What made these supports meaningful was the personal connection and fit with their own needs.
  There were a few moments where fathers/non‐birthing partners felt seen, heard, and included in options for support even if it encompassed support for the parenting dyad, not specifically just the father/non‐birthing parent such as participant 1: ‘*the main doctor that we had at the fetal medicine unit, she would always check in and see if we had a support base and stuff at home, same with the midwives. They did offer a lot of family support’*.
b. **Seeking whole‐person support**
  Fathers/non‐birthing partners expressed a desire for support that extended beyond the clinical aspects of TFMR, encompassing workplaces, within the community and family and friend dynamics. Rather than isolated medical interventions, they sought integrated care that recognised the emotional and social dimensions of the whole experience, including processing grief and adjustment after the loss. This was articulated by participant 12 who did not ‘*think there was enough care or understanding from the medical profession for the grief side of it and the explanations and even going down the avenue of, have you got support in your family and how are you telling family’* and echoed by participant 11 who felt ‘*everyone was so amazing that if I think back on this situation, I would still feel really supported, but not supported in a mental health way’*. There were several situations along the TFMR journey that whole‐person care was highlighted as important, particularly during the waiting and decision‐making periods.
  The waiting period was a specific time mentioned by most of the fathers/non‐birthing partners where support was missing, primarily to address the emotional dimensions of the waiting. The ‘waiting period’ is a significant aspect, often followed by the initial ultrasound or prenatal testing and then stretching across subsequent medical appointments and further tests, often lasting for weeks or months. This was described by participant 11 who stated ‘*I'm pretty sure we didn't have any support because we went home and we were like, oh my God, what shall we do? We waited for a couple of hours and then we called the clinic where we did the scan. They said, we referred it to the hospital, or to your GP, so you just need to wait… then we called our GP, but she was also saying you just need to wait. We went crazy that weekend’.*
  A lack of touch points with health services during this time reduced opportunities for fathers/non‐birthing partners to access support, particularly as they were not regarded as the ‘patient’ and were therefore unlikely to be contacted by health professionals to check on their wellbeing.
  ‘You've just got to wait your time; we were told there's nothing we could do, we just had to make that next appointment. So, by the time we got into the specialist, we were probably four or five weeks into the waiting. And during that time, we'd only seen the GP once, which was the initial consultation’.
  Participant 5
  The waiting period highlights the desire for whole person support across the TFMR journey. While medical pathways were proceeding appropriately, the absence of structured contact and clear avenues for support during a time of uncertainty left many fathers/non‐birthing partners feeling they had to self‐source resources and tools to cope. The exception to this experience was participant 6 who, serendipitously, found themselves in the right place to access support and medical care*: ‘the day we found out, we had a midwife appointment, and she put us in touch with a social worker straight away. Like we got really, really lucky with that midwife appointment because she literally called the doctor and then quickly put a rush on things*’.
  The decision‐making period involved silent struggles’ which captured the way couples were left to shoulder the immense weight of decision‐making after receiving news that something might be wrong with their baby without much support except for each other. While medical teams provided the clinical information, parents often described a lack of emotional or relational support to help them process what the information meant and how to move forward. Fathers/non‐birthing partners reflected on how little support or acknowledgement there was of the lifelong consequences of these decisions, which continued long after the medical procedures were complete.
  Participants consistently indicated that support to assist during the decision‐making process was minimal, particularly for navigating communication as a couple: ‘*we weren't given support as a couple to make the decision*. *The support we received was again through* [the midwife]’ (Participant 5). Despite this, many couples described ‘being on the same page’ and drawing on their own communication skills to navigate difficult discussions. Participant 11 reflected: ‘*We were actually really lucky that we were on the same page most of the time. Of course we were not always on the same page, but we had this really good phase of our relationship that we were able to talk with each other’*.
  For some, the sense of being a team was crucial, with the couple checking in with each other and taking it in turns to support each other, such as participant 2: ‘*we've been such a team through the whole thing. Like being there, letting each other be the more emotional one at times, you know, like giving each other turns’.* This was echoed by participant 3 who described ‘*that one person stood up that day or in that moment or that half day, whatever it was, and just really carried each other*’.
  While most couples processed decisions together, some worked through them individually, with fathers/non‐birthing partners sometimes reaching a decision earlier than their partners. Participant 1 explained: ‘*I kind of had a feeling that's where it was all going, but I wasn't gonna push just to make her choose either… when she finally came around to it, then yeah, we talked about it again and cried so much’.* Participant 12 described differences in how they navigated decision‐making: ‘*My wife and I are very different in our approaches. She was sort of just completely shut down, and I wanted to find the answers. So, I joined Facebook groups, I read papers, I spoke to people online’*.
  Participants acknowledged the fragility of this alignment, recognising how differently the process could unfold if they had opposing views. Participant 3 reflected: ‘*I think we were very lucky to be on the same page. But it could be so different if XXX* [partner] *was strongly for and I was strongly against. That can make and break relationships’*.
  Several participants highlighted the lack of acknowledgement about and support for decisions made in moments of difficulty that echoed across their lives. Participant 1 shared: ‘*I don't think the hospital really said these are things that you're gonna have to constantly think about or have weighing on your mind. You get to make a decision which is a crappy decision that you don't want to make a choice on, and then you get to carry that forever with you’.* For others, regret and doubt surfaced quickly after birth. Participant 5 recalled: ‘*I vividly remember going, what have I done? What have we done? If she could survive that* [birth]*… we always have that sense of doubt’.* The information about carrying the weight of the decision over time was generally brought up by community pregnancy loss support services, and it was not clear what supports fathers/non‐birthing partners were using to help them learn to carry the weight of the decision.
  Other areas where whole‐person care was identified as important were access to mental health care, workplaces and support from family and friends. By the time fathers/non‐birthing partners were in a place to acknowledge and tend to their own mental health, they found the process of reaching out for help challenging such as participant 2 who felt ‘*when you are in a place of absolute crisis, trying to find a therapist and get a mental health plan is such a barrier… those processes, they're not realistic for people in crisis at all’*. The effort required to reach out to someone, especially without an existing therapeutic relationship, was an obstacle: ‘*I think it's already hard to make an appointment with someone but then calling someone not knowing who is on the phone, I think that's another hurdle. You need even more strength to call a number and not know what happens after that call*’ (Participant 11).
  The importance of an existing therapeutic relationship or a known and trusted conduit to connect fathers/non‐birthing partners to supports was highlighted with several participants describing how peers with lived experience of TFMR became sources of guidance and comfort such as Participant 1: ‘*We were lucky. One of my friends, her sister had gone through the same thing … So, I had that opportunity to have a bit of a chat to someone. Yeah, so that was actually, surprisingly really helpful’*. These connections often provided practical advice and emotional reassurance, delivered in a way that felt accessible and relatable because of existing trust. Participant 4 described the benefit of such a relationship: *‘One of my friends went through the same thing the year before us. She jumped on the phone with us and ran us through everything that she'd been through, and it was very, very useful and she was a good asset to have. There's that pre‐existing relationship between us and her, like we're already friends and feel comfortable speaking to her’*.
  As part of whole‐person support, participants indicated that support from their workplace was vital. One father/non‐birthing partner described the challenges returning to work after the birth: ‘*She* [boss] *made me go back to work nine days after and there were no ifs, no buts. I said to her, mentally I'm not ready and my wife needs me at home, and she said I'll see you Monday. I kind of read between the lines and if I didn't show up Monday I didn't have a job’* (Participant 3).
  In contrast, many workplaces navigated the balance of work leave entitlements, the business needs, and the needs of the father/non‐birthing parent in a way that made them feel supported and valued. For participant 4, this was cohesive support from colleagues and management to facilitate their needs at the time: ‘*All my workmates sort of got in touch and offered support. The health and welfare branch of work rang me up when I booked my sick leave and said, “is there anything we can do to help?” I explained the circumstances to them and they were like, here's all the services we have available, do you want me to start arranging any of those?*’ For participant 9, this was demonstrated in the way their workplace found them a new role following the loss: ‘*I had four months off and my work was good and then I was basically like I can't go back to doing what I was doing before and so they were like yep, we'll find you something and they did’*.
  Friends and family also played an important role in whole‐person support, although this support varied in quality and consistency. When it was present, fathers/non‐birthing partners valued support that was non‐judgemental and allowed them to grieve authentically. As participant 6 explained, ‘*we got fortunate that all our families accepted our decision. None of them questioned it or challenged it or anything. They held our hands and watched us cry and did what they could and cried themselves*’. However, many participants also shared experiences of friendships and family relationships changing in the aftermath of their loss. Some close relationships weakened when support was absent or short‐lived. Participant 2 reflected, ‘*It was interesting though, through that process, kind of seeing other friendships that you thought were really close and those people not stepping up….and even family, some close family that you thought would be there, but who weren't really’.*
c. **Partners as a support rather than being supported**
  Most fathers/non‐birthing partners described taking on the role of being the supporter, particularly during labour and birth. In this role, they often prioritised their partner's needs above their own, setting aside their own emotions and wellbeing: ‘*I just felt I had to step up. My role was to support XXX* [partner]*. She'd physically delivered him… I felt it was my role as a husband to step up and focus on her and look after her. I probably pushed my emotions aside a little bit and didn't do enough to focus on my wellbeing’.* (Participant 3).
  This sense of responsibility often extended beyond the hospital, with some participants framing themselves as primary caregivers once they returned home: ‘*The moment we walk out that door* [hospital door], *I am the care provider for XXX* [partner] *until she's back on her feet…. I had to do what I needed to do to make sure she was okay’* (Participant 6).
  Participants also highlighted an underlying belief that support was a scarce resource, particularly within hospital settings, and should be reserved for the birthing parent. As one put it: ‘*I got zero support. If there is a finite amount of support and things available, then I would gladly give them to XXX* [partner]’ (Participant 8). Others described a fear that seeking/accepting care for themselves would detract from their partner's treatment: ‘*if you complain and then the staff get a bit shitty…. you obviously don't want XXX's* [partner] *care to be compromised in any way, shape or form. So, you stay silent’* (Participant 2). Silence and suppression of their own grief were seen as acts of protection, with non‐birthing partners framing emotional restraint as a way of avoiding burdening their partner further: ‘*I didn't allow myself to have that* [moment] *because I didn't feel like I could, I guess. Because if I did that, it would just be at the detriment of XXX* [partner]’ (Participant 4).
  As part of supporting their partner, fathers/non‐birthing partners expressed a wish to feel useful and involved during the TFMR experience. Several fathers/non‐birthing partners wanted support that enabled them to contribute meaningfully, particularly in ways that relieved pressure on their partners. As participant 6 explained: ‘*We like to be useful. We want to be helping. We want to have a job*, *even if they invented some crappy little job….Just give me something to focus on that feels like I'm contributing’.* This sense of wanting to participate was especially salient in situations where fathers/non‐birthing partners could have undertaken practical or administrative tasks: ‘*There was a stack of paperwork… and not a single thing could be filled out by me. It all had to be filled out by XXX* [partner]. *She was hours after delivering a baby, she was tired, she was exhausted, she was emotionally gone … and I was like, please, just give me this. I know all of these answers’* (Participant 5). This also included a desire for the fathers/non‐birthing partners to co‐sign the medical termination paperwork. Currently, in Australia, the birthing partner must sign the paperwork for the medical process of termination, with fathers/non‐birthing partners not being permitted to co‐sign. The desire to co‐sign the paperwork was driven by fathers/non‐birthing parents wanting to share the burden placed on their partners, have their presence recorded, and to have their solidarity as a couple acknowledged through the presence of both parents’ names as described by participant 9: ‘*And that hurt a lot yep because like I was like oh, they've just put it all on the XXX* [birthing partner] *and like you know it doesn't need to be some legal thing, but just as long as my name's on there next to XXX so it shows that yeah, I was there, and we're there together’*. Conversely, other participants experienced being given tasks as burdensome, reinforcing a sense of being peripheral to support and care, rather than the recipients of care: ‘*Because they look at you like, oh, XXX* [partner] *is bleeding, help her out of the bath, go get that cloth. Like, it's almost like you're there just for extra hands, you know*’ (Participant 8).

## 3 Discussion

This study explored fathers’ and non‐birthing partners’ perceptions and experiences of support during a TFMR. The overarching theme, *support in the shadows*, captures how non‐birthing partners largely felt peripheral within systems of care and wider social support networks throughout the TFMR experience. Participants described a pattern of silent responsibility, emotional invisibility and a lack of meaningful recognition of their needs. Together, these findings highlight gaps in support for fathers/non‐birthing partners and underscore the need for more inclusive, partner‐responsive models of TFMR care.

An important finding from the study, which appears to be more specific to the TFMR experience than other forms of pregnancy loss, was participants’ candid reflections on the enduring weight of making the decision to end a wanted pregnancy and its ongoing impact. Although previous research has shown that grief and other psychological sequelae following TFMR may persist for many years, reports of decision regret are low [26, 27, 28, 29, 30], there is limited evidence regarding the types of support that help parents live with this decision in the months and years following TFMR. This represents an important opportunity to strengthen care for both fathers and non‐birthing partners, as well as birthing parents. Given that parents information needs change over time and details provided during periods of acute distress may not be fully retained, healthcare services could consider providing written information alongside verbal discussions, enabling parents to revisit the medical information and diagnosis when needed. Models of care that provided opportunities for follow‐up, ongoing questions and reassurance regarding the diagnosis and prognosis may help parents carry the load of TFMR while acknowledging that grief and uncertainty often evolve over time. Further research is needed to better understand how parents experience and carry the weight of decision‐making in a TFMR journey and to identify interventions that support families throughout this lasting process.

Our findings are consistent with previous qualitative pregnancy loss research demonstrating that partners often adopt roles of protector, advocate and emotional anchor during pregnancy loss [9, 10, 15, 26, 31, 32]. There have been several recommendations in the research for recognition of father's/non‐birthing partners as individuals with their own support needs [33]; however, practices are often slow to change. One aspect of taking on the supporter role that does not appear to be explored in the qualitative research so far is the insight that fathers/non‐birthing partners perceived support to be a scarce resource, particularly within hospital environments, and that support should be directed to the birthing parent, highlighting a key clinical challenge. Healthcare staff should explicitly reiterate to fathers/non‐birthing partners that support offered is not finite or resource constrained.

Participants also described feeling invisible, and that support was fragmented across the TFMR journey with a desire for whole‐person support. Research indicates that the invisibility that partners feel during labour and birth is not uncommon, regardless of the birth outcome [34, 35, 36, 37]. Research also shows that the exclusion of fathers from maternity and postpartum care is a system‐wide issue related to institutional practices, service design and the culture of maternity services [38]. While there are improvements to be made in supporting fathers/non‐birthing partners during labour and birth, participants in our study specific to TFMR also indicated there were notable gaps before and after hospital care, particularly during the waiting period, the decision‐making, and after the procedure. Similar desires for continuity of care have been described across the broader pregnancy loss literature [7, 31]. Participants’ accounts indicate a desire for support that does not conclude with the procedure but encompasses preparation for decision‐making, care during waiting periods, acknowledgement of grief and access to ongoing psychological and peer support. An approach that includes sustained emotional and relational support for all members of the family in a TFMR journey emphasises not only access to essential reproductive healthcare but also the conditions that enable individuals and families to experience that care with dignity, support and autonomy [2].

Importantly, our findings highlight gaps in research and practice for marginalised groups, not just for fathers, but for same‐sex couples and gender‐diverse non‐birthing parents. Notably, qualitative research rarely includes non‐birthing partners in same sex or gender‐diverse relationships, leaving a significant gap in understanding the full diversity of partner experiences. Culturally and linguistically diverse individuals, those in rural locations, and those with low socioeconomic status experiences also remain largely absent from the TFMR and support research. Designing research studies that explicitly include all non‐birthing parents, regardless of gender, identity, or family structure, is necessary for ensuring support is individualised and meets non‐birthing parent's needs.

### 3.1 Strengths, Limitations and Future Directions

This study contributes needed insight into the support needs of fathers/non‐birthing partners throughout the TFMR experience. Findings are shaped by the characteristics of the sample and offer in‐depth insights into a small group of participants. Further research with larger and more diverse samples is needed to capture the full diversity of fathers/non‐birthing partner experiences, acknowledging that individual needs may still differ. Participants self‐selected into the study, and the perspectives of those who do not seek support or who have limited access to TFMR‐specific services remain understudied. Future research should explore tailored co‐designed support pathways and interventions for fathers/non‐birthing partners and examine cultural and gender‐diverse experiences. Furthermore, research should explore how professional perspectives may contribute to or mitigate the TFMR experience to enable implementable strategies to improve healthcare practices for TFMR.

## 4 Conclusion

Fathers/non‐birthing partners are often under‐supported as they navigate an emotionally and morally complex journey ‘*in the shadows*’ during and after a TFMR. Recognising and addressing support for fathers/non‐birthing partners in this space is greatly needed to develop compassionate and inclusive care that honours the grief, needs and roles of all parents.

## Author Contributions

**Sarah Fogarty:** conceptualisation, methodology, formal analysis, resources, data curation, writing – original draft, writing – review and editing, visualisation, project administration. **Kirstin Tindal:** conceptualisation, methodology, formal analysis, writing – review and editing. **Alexander E. P. Heazell:** conceptualisation, methodology, writing – review and editing. **Niki Munk:** conceptualisation, methodology, writing – review and editing. **Frances M. Boyle:** conceptualisation, methodology, writing – review and editing. **Phillipa Hay:** conceptualisation, methodology, writing – review and editing, supervision, project administration.

## Funding

The authors have nothing to report.

## Ethics Statement

The study was conducted in accordance with the Declaration of Helsinki and approved by the Human Research Ethics Committee of Western Sydney University (No: H15715). The Australian HRECs are the gatekeepers ensuring research follows the ethical blueprint established by the Declaration of Helsinki and detailed in the National Statement.

## Consent

Informed consent was obtained from all participants in the study. All study participants gave informed written consent for the interview and the use of their data.

## Conflicts of Interest

Sarah Fogarty is a practicing massage therapist. Niki Munk is a full‐time academic, long‐time consultant and mentor to Sarah Fogarty, who receives no financial compensation for her effort on this work and is Research Director of the Massage Therapy Foundation. It is not expected that the study findings will yield any financial gain for Sarah Fogarty or Niki Munk. Niki Munk's role at the Massage Therapy Foundation has no bearing on the study implementation or results. Phillipa Hay has received sessional and lecture fees from the Australian Medical Council, Therapeutic Guidelines publication, and New South Wales Institute of Psychiatry and royalties from Hogrefe and Huber, McGraw Hill Education, and Blackwell Scientific Publications, and she has received research support from CAPES, NHMRC and ARC. She is the Chair of the National Eating Disorders Collaboration in Australia (2019‐current). She has been a consultant for Tryptamine Therapeutics Pharmaceuticals. Kirstin Tindal is employed by The Stillbirth Centre of Research Education in a research capacity.

## Supporting information


Supporting File

## Data Availability

The qualitative datasets generated and/or analysed during the current study are not publicly available due to the nature of the interviews and the high risk of identification of study participants and/or healthcare providers and healthcare institutions. These data are available from the corresponding author on request.
