# Supplementary material for: Support in the Shadows: Findings From a Qualitative Study Exploring Fathers’ and Non‐Birthing Partners’ Perceptions and Experiences of Support During a Termination for Medical Reasons
Source: Health Expect. 2026 Aug 2;29(4):e70799. doi: 10.1111/hex.70799 (PMC13429099; doi:10.1111/hex.70799)
Supplement: Supplementary file 1 — Supporting File [file HEX-29-e70799-s001.docx]

***Semi-structured interview questions***

*Confirm consent for interview to be recorded.*

*Support services*

*1. Beyond Blue 1300 22 4636 24 hour support for women and men.*

*2. SANDS 1300 072 637 24 hours helpline.*

*3. Lifeline 13 11 14 (24 hours service).*

*4. Own healthcare professional of choice (i.e. past treating physician)*

*Start with something along the following:* Thank you for meeting up with me today to share your experience of support during your TFMR journey. Please only share information that you feel comfortable to share and if you don’t want to answer a question, please say pass or let me know however you need to.

I’d like to ask you some questions about your experience of support during your TFMR journey. I am going to break it down into stages so please let me know if a stage is not relevant to your experience or if there is a part of the TFMR journey that I don’t ask about.

*Reiterate that if one thing in on part of the journey links to another part of the journey then they don’t have to wait till that part of the journey is brought up, they can share wherever we are at.*

**Let’s start with the beginning.** Tell me about how and when you found out that things might not be okay for your little one.

Tell me about any support you received at this stage and what did it look like.

Tell me about what support you would have liked at this point.

Tell me if you received any support that was unhelpful (either said or offered)

**Let’s move into the next part of your journey. Did you undergo further testing?**

If so, tell me about any support you received at this stage and what did it look like.

Tell me about what support you would have liked at this point.

Tell me if you received any support that was unhelpful (either said or offered)

**Let’s move into the next part of your journey: Making decisions.**

Tell me about the time of making a decision.

Tell me about the process of making decisions for your family and how that was for you.

Tell me about any support you received at this stage and what did it look like.

Tell me if you received any support that was unhelpful (either said or offered)

Did you feel like you were supported regardless of what decision you made (i.e., continue with the pregnancy and allow your baby to come when they wanted or terminating the pregnancy)

**Let’s move to the next part of the journey and that is preparing for the birth/termination.**

Tell me about what support you received between making the decision and the birth/termination of your baby.

Tell me about what support you would have liked at this point.

Tell me if you received any support that was unhelpful (either said or offered)

Tell me about any support you received to prepare for the birth/termination of your baby.

Tell me about what support you would have liked at this point.

Tell me if you received any support that was unhelpful (either said or offered)

**Let’s move into the next part of your journey: The Birth/termination of your baby.**

Tell me about any support you received at this stage and what did it look like.

Tell me about what support you would have liked at this point.

Tell me if you received any support that was unhelpful (either said or offered)

*Include in this section the hospital stay.*

**Let’s move into the next part of your journey: Coming home without a baby.**

Tell me about any support you received at this stage and what did it look like.

Tell me about what support you would have liked at this point.

Tell me if you received any support that was unhelpful (either said or offered)

**Let’s talk about the 6-12 months and onwards after you birthed your baby:**

What things did you find difficult during this time (if need a prompt things like, not being able to share with others about what happened, not knowing what is on your birth certificate if you got one (e.g. TMFR or stillbirth or miscarriage), not feeling like they belong, anniversaries etc.

Tell me about any support you received at this stage and what did it look like.

Tell me about what support you would have liked at this point.

Tell me if you received any support that was unhelpful (either said or offered)

*Include in this section time after 12 months if appropriate.*

**If you had a pregnancy after your TFMR please tell me about the support your received during the pregnancy after a loss**

Tell me about what support you would have liked at this point.

Tell me about what support you would have liked at this point.

Tell me if you received any support that was unhelpful (either said or offered)

Thank them for their time and reiterate support services if they need them.

Remember to ask about the different types of support that could be offered or not e.g., health care support, procedural support, home support, emotional support, support services, self-care support, connection support, social circle support etc.
